# L-tetrahydropalmatine attenuates ketamine reward effect via modulating the miR-27a-3p/MAP2K4 axis

**DOI:** 10.3389/fgene.2026.1701742

**Published:** 2026-06-29

**Authors:** Yan Du, Xing-Cui Gao, Qing Ma, Bei Li, Yanting Chen, Hong-Liang Su, Li Du, Tai-Gang Liang

**Affiliations:** 1 School of Pharmacy, Shanxi Medical University, Taiyuan, Shanxi, China; 2 School of Forensic Medicine, Shanxi Medical University, Taiyuan, Shanxi, China; 3 The Cancer Hospital of Shanxi Province, Taiyuan, Shanxi, China

**Keywords:** BDNF-TrkB, ketamine reward effect, L-tetrahydropalmatine, MAP2K4, MiR-27a-3p

## Abstract

**Introduction:**

Ketamine (KET) addiction has already been a serious problem all over the world, which could induce neurological and psychological harm. Levo-tetrahydropalmatine (l-THP), a major alkaloid extracted from the Chinese medicinal plants *Corydalis* and *Stephania*, has been shown to attenuate ketamine (KET) induced conditioned place preference (CPP) in rats. Nevertheless, the precise mechanism remains unknown, and further research is necessary.

**Methods:**

This study aimed to investigate the role of the brain-derived neurotrophic factor/tropomyosin-related kinase B (BDNF/TrkB) signaling pathway and microRNAs (miRNAs) in the modulatory effects of l-THP on KET reward effect. A rat CPP model and PC12 cell addiction model were developed. Next-generation high-throughput miRNA sequencing was utilized to identify candidate miRNAs. Behavioral assessments, real-time PCR, Western blotting, and cell transfection studies were conducted to clarify the impact of the selected miRNAs and the mechanism of l-THP intervention on KET reward effect.

**Result:**

l-THP effectively attenuated KET-induced CPP and activated key proteins in the BDNF/TrkB signaling pathway. In addition, l-THP reversed the increased expression of miR-27a-3p in KET-abused rats and PC12 cells. Furthermore, inhibition of miR-27a-3p expression using an miR-27a-3p inhibitor in PC12 cells resulted in increased mitogen-activated protein kinase 4 (MAP2K4) expression, indicating that MAP2K4 is a potential functional target of miR-27a-3p *in vitro*.

**Conclusion:**

Our findings demonstrate that l-THP attenuates KET reward effect by modulating the BDNF/TrkB pathway and the miR-27a-3p/MAP2K4 axis, which may be promising targets for the intervene of KET reward effect.

## Introduction

Over the past few decades, the non-medical use of ketamine has surged globally, escalating into a severe public health crisis that imposes substantial burdens on individuals, economies, and societies (Bokor and Anderson, 2014). In China, ketamine (KET) has emerged as the third most prevalent abused substance, trailing only heroin and methamphetamine (National Narcotics Control Commission, 2022). Research has consistently demonstrated that the mesolimbic dopamine (DA) system plays a pivotal role in mediating drug-induced behavioral and neuronal alterations (Volkow et al., 2009). Rodent studies have revealed that KET administration acutely elevates DA levels in the nucleus accumbens (Lüscher and Ungless, 2006; Rocha et al., 1996; Suzuki et al., 2000; De Luca and Badiani, 2011; Zanos et al., 2017). Consequently, KET addiction is characterized by high relapse rates following withdrawal (Thomas and Chambers, 2025), yet no pharmacologically effective treatments currently exist to mitigate this risk (Roberts et al., 2024).

Given these challenges, both medical professionals and society urgently require a deeper mechanistic understanding of KET reward effect and the development of targeted therapeutic interventions. The mesolimbic dopamine (DA) system plays a critical role in drug abuse, as it mediates reward-related behaviors and drug-seeking motivation. Brain-derived neurotrophic factor (BDNF), a key regulator of neural plasticity, enhances the adaptability of neural and behavioral functions through its modulatory effects on the mesolimbic DA system (Duman, 2002). By influencing synaptic plasticity and cellular processes essential for learning and memory (Duman, 2002), BDNF may exert profound effects on the development of drug addiction. Specifically, BDNF modulates DA release and addictive behaviors via its receptor tropomyosin-related kinase B (TrkB) (Liang et al., 2011), suggesting a potential therapeutic target for addiction intervention. However, the precise molecular mechanisms underlying ketamine (KET) reward effect remain unclear, particularly the changes induced by levo-tetrahydropalmatine (l-THP) treatment. To address this gap, we focused on the hippocampus—a limbic system structure pivotal for knowledge acquisition, memory retention, emotional processing, and conditioning. Given its integration with the mesolimbic DA system and BDNF signaling pathways, the hippocampus represents a critical region for investigating KET reward effect and l-THP-mediated therapeutic effects.

In recent years, accumulating research has focused on the regulatory role of microRNAs (miRNAs) in drug addiction (Smith and Kenny, 2018; Wang et al., 2022; Zhu et al., 2022). As endogenous non-coding RNA molecules with 18–25 nucleotides, miRNAs post-transcriptionally regulate gene expression by promoting mRNA degradation and inhibiting translation, thereby serving as negative feedback regulators of mRNAs (Lindow and Gorodkin, 2007; Barbato et al., 2008). Notably, the enriched expression of certain miRNAs in the brain suggests their involvement in neuronal morphogenesis, addiction-related behaviors, and memory consolidation (Ashraf et al., 2006). Given that ketamine (KET) induces significant alterations in miRNA expression within brain regions implicated in addiction (Li et al., 2018), these molecules may serve as critical mediators of KET reward effect. Specifically, miRNAs could regulate the mesolimbic dopamine (DA) system and BDNF signaling pathways—both of which are central to addictive behaviors and synaptic plasticity. Therefore, elucidating the regulatory mechanisms of miRNAs in KET reward effect is essential for unraveling the condition’s molecular underpinnings and identifying novel therapeutic targets.

**FIGURE 1:**
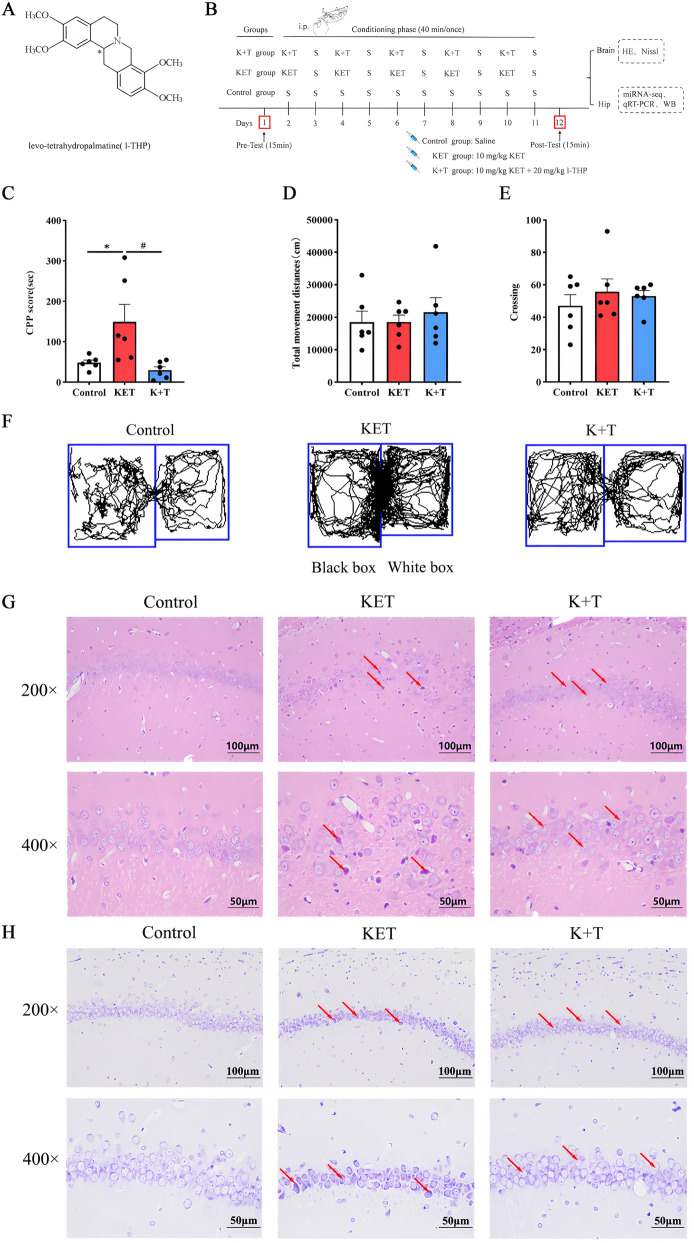
**(A)** Chemical structure of l-THP. **(B)** Diagram illustration of the CPP test. **(C)** Effects of l-THP on time spent in the white compartment. **(D)** The total distance traveled by rats in each group in the white box after training. **(E)** The number of shuttles by rats in each group in the white box after training. **(F)** Visual representations depicting the trajectories followed by rats within the CPP compartments. **(G)** HE staining of brain tissues. **(H)** Nissl staining of brain tissues. Data are expressed as mean ± SEM (*n* = 6 per group). *p < 0.05 KET group vs. Control group; ^#^ *p* < 0.05 K + T group vs. KET group.

Tetrahydropalmatine (THP), a primary active component derived from *Corydalis ambigua* and *Stephania tetrandra*, exists in two optical isoforms: the l-enantiomer (l-THP) and d-enantiomer (d-THP) (Wang and Mantsch, 2012). Notably, only l-THP (Figure 1A) exhibits central analgesic, sedative, and stabilizing effects, and its purified form has been clinically utilized as a pain reliever and sedative in China for over 4 decades (Liu et al., 2021). l-THP has garnered significant attention due to its potent central nervous system effects, particularly as a noncompetitive antagonist of dopamine D1 and D2 receptors (Chu et al., 2008) and its modulatory effects on dopamine D3 receptors (Mantsch et al., 2007; Xi et al., 2007). Although our prior work demonstrated that l-THP attenuates ketamine (KET)-induced conditioned place preference (CPP) in rats (Du et al., 2017) and has pharmacokinetics effects on KET in rat plasma (Du et al., 2020), the exact molecular mechanism underlying its inhibitory effect on KET reward effect remains unclear. Given that KET reward effect is associated with alterations in miRNA expression within brain regions implicated in addiction (Chandrasekar and Dreyer, 2009), it is critical to investigate whether l-THP treatment modulates miRNA levels in the hippocampus—a key structure involved in memory and addiction-related behaviors. However, the expression profiles of miRNAs in the hippocampus of KET-reward effect rats and their alterations following l-THP treatment have not yet been documented.

In this research, our objective was to investigate the function of BDNF/TrkB signaling pathway and miRNA expression profile in the hippocampus of rats with KET-induced CPP and elucidate the potential underlying mechanism of the inhibitory effect of l-THP on KET reward effect through the screening, identification, and validation of target miRNAs and their target genes in an *in vitro* setting.

## Materials and methods

### Chemicals and reagents

l-THP (99.00%) was purchased from Sigma-Aldrich Inc. (USA). KET hydrochloride was obtained from Heng-Rui Pharmaceutical Factory (Jiangxi, China). RPMI 1640 medium were purchased from Gibco (Grand Island, United States); Fetal bovine serum, trypsin, penicillin, and streptomycin were purchased from Pricella (Wuhan, China); RT-qPCR reagents were purchased from Mei5bio (Beijing, China); The bicinchoninic acid (BCA) protein assay kit, radioimmunoprecipitation assay (RIPA) buffer, and antibodies against MAP2K4 (A01725-1) and BDNF (PB9075) were purchased from Boster (Wuhan, China); β-actin (AC038), TrkB (A21227), PI3K (A21227), p-PI3K (AP0427), CREB (A10826), and p-CREB (AP0019) were purchased from ABclonal (Wuhan, China); AKT (10176-2-AP) and p-AKT (28731-1-AP) were purchased from Proteintech (Wuhan, China); ERK1/2 (4690) and p-ERK1/2 (4370) were purchased from Cell Signaling Technology (Boston, United States); ECL reagent was purchased from MeilunBio (Liaoning, China); The MTT assay kit was purchased from SolarBio (Beijing, China). MiR-27a-3p inhibitor was purchased from Hanbio (Shanghai, China).

### Animals

Male Sprague-Dawley (SD) rats (6–8 weeks) were purchased from SPF Biotechnology Co., Ltd. Animal license number: (SCXK [BeiJing] 2019-0007). All animals were housed in a controlled environment with a 12-h light and dark cycle (8:00–20:00 light, 20:00–8:00 dark), the CPP experiment was conducted from 8:30 to 18:30. The animals were maintained at a temperature of 22 °C ± 2 °C and humidity of 55% ± 5%. They had unrestricted access to food and water and underwent a 1-week acclimation period. Approval for all animal-related experimental procedures was obtained from the Institutional Animal Care and Use Committee of Shanxi Medical University. Thirty rats underwent the pre-test. To eliminate major individual differences, we excluded those rats that spent over 600 s in the session in either compartment or crossed less than 20 times between the compartments (Nine rats were excluded, and three rats died). Eighteen rats were divided into three groups: (1) a control administered saline (n = 6, Control group); (2) KET treatment (n = 6, 10 mg/kg, KET group); and (3) l-THP (20 mg/kg) + KET (10 mg/kg) treatment (n = 6, K + T group). The treatments were administered by intraperitoneal injection. l-THP was administered 30 min before KET administration. After the CPP post-test on day 12, three rats in each group were randomly selected. They were anesthetized by isoflurane and sacrificed by cervical dislocation immediately, and their whole brains were removed, the hippocampal tissue was separated on the ice plate, and frozen at −80 °C. The hippocampal tissue is used for subsequent Western blot and PCR experiments. The other three rats in each group were randomly selected. The whole brain of them were removed. They were fixed in 4% paraformaldehyde and embedded in paraffin for the histopathological examination, nissl staining, and immunohistochemistry experiment.

### CPP model

The CPP apparatus (JLBeHv, China) consists of two equally sized compartments (L × W × H: 30 × 30 × 40 cm), as described previously (Du et al., 2021). Eighteen qualified rats in the baseline test were randomly divided into three groups: Control, KET, and K + T group. In our previous study, we examined the impact of varying doses of l-THP and found that 20 mg/kg of l-THP significantly attenuated KET-induced CPP in rats (Du et al., 2017). Therefore, in this study, a dose of 20 mg/kg of l-THP was applied.

On day 1, the rats were placed in CPP equipment for 15 min, and they were allowed to move freely within the compartment. On days 2, 4, 6, 8, and 10, the rats received an intraperitoneal injection of KET (10 mg/kg) in the KET group or the same volume of saline in the control group, following this, they were promptly isolated in the white chamber for a period of 40 min. In addition, on the same days (days 2, 4, 6, 8, and 10), rats in the K + T group received an intraperitoneal injection of l-THP 30 min before KET, following this, they were instantly confined to a white box for a duration of 40 min. In each group, all rats received an equal volume of saline on days 3, 5, 7, 9, and 11. They were then placed in the black box for 40 min. The post-test is day 12. The rats were recorded on video during a 15-min trial, and the paths of their movements were recorded and evaluated using the DigBehv analysis system (Figures 1B,F).

### Histopathological examination, Nissl staining, and immunohistochemistry

Rat brains were fixed in 4% paraformaldehyde and embedded in paraffin. After 24 h, the samples were sectioned for further analysis. Hematoxylin and eosin, as well as Nissl staining, were performed on the brain sections. Samples were deparaffinized using an environmentally friendly dewaxing solution, dehydrated in ethanol, and stained with purple tar. After dehydration, clearing, and mounting, neuronal damage was examined. Images were captured using a light microscope (Olympus, Tokyo, Japan). Primary antibodies were diluted to suitable proportions in PBS (BDNF, 1:1200; p-ERK, 1:100; p-CREB, 1:1000). The expression level was quantified using ImageJ 1.54j. H-score = strong positive percentage × 3 + medium positive percentage × 2 + weak positive percentage × 1.

### Western blot analysis

Rat hippocampal tissue and PC12 cells were lysed in RIPA buffer. Subsequently, the separated tissue lysates were transferred onto PVDF membranes for protein blotting using SDS-PAGE. The antibodies used are as follows: MAP2K4 (1:1000), BDNF (1:1000), TrkB (1:15,000), PI3K (1:1500), p-PI3K (1:700), AKT (1:7000), p-AKT (1:2000), ERK1/2 (1:1000), p-ERK1/2 (1:1000), CREB (1:1000), p-CREB (1:1000), and β-actin (1:40,000) at 4 °C overnight. An ECL solution was prepared to visualize the protein bands, and ImageJ 1.54J software was used to quantify the images.

### Cell culture and exposure to KET and l-THP

PC12 cells were obtained from Wuhan Pricella Biotechnology Company. Cells were seeded at a density of 1 × 10^5^ cells/mL in 96-well plates (100 μL per well), and then randomly assigned to three groups: Control, KET (1 mmol/L), and K + T (KET, 1 mmol/L, l-THP, 25 μmol/L). We examined the impact of various concentrations of KET and l-THP on the viability of PC12 cells. As a result, we chose 1 mmol/L of KET as the model dose and 25 μmol/L of l-THP as the treatment dose. A total of 1.25 × 10^5^ cells were seeded in 12-well plates. Cells in the K + T treatment group were pre-incubated with l-THP for 15 min before KET exposure.

### miRNA sequencing and prediction of target genes

Hippocampal miRNA-seq was performed using next-generation high-throughput sequencing. An RNA library was created using DNBSEQ technology. Next-generation miRNA sequencing data were analyzed for each array. The selection criteria were set as *P* < 0.05 and fold change >2 or fold change <0.5 to identify differentially expressed miRNAs. Gene function was annotated by utilizing the Gene Ontology (GO) database and the Kyoto Encyclopedia of Genes and Genomes (KEGG) (Li et al., 2018). To forecast and examine the genes targeted by the miRNAs that exhibit differential expression, we conducted a search across three primary miRNA target gene prediction databases: TargetScan (http://www.targetscan.org/), miRDB (https://mirdb.org/), and miRanda (http://mirtoolsgallery.tech/mirtoolsgallery/node/1055).

### Quantitative real-time PCR (qPCR)

RNA was reverse-transcribed and quantified by qPCR using a LightCycler 96 instrument (Roche, Switzerland). The M5 miRNA qPCR Assay Kit and 2 M5 HiPer SYBR Premix EsTaq were used to quantify miRNA and mRNA. The PCR protocol to quantitate miRNA was as follows: 94 °C for 2 min, followed by 45 cycles at 94 °C for 10 s, with a final recording step at 60 °C for 30 s. Small nuclear RNA U6 was used as an internal control. The mRNA quantitative PCR protocol was as follows: 95 °C for 30 s, followed by 40 cycles at 95 °C for 5 s and 60 °C for 30 s, 95 °C for 5 s, 60 °C for 30 s, 95 °C for 1s, and 50 °C for 30 s. GAPDH was used as the internal control, and the 2^−ΔΔCt^ formula was used to calculate gene expression. All primer sequences of miRNAs are listed in Table 1.

**TABLE 1:** PCR primer sequences of miRNAs.

miRNA	PCR primer
miR494-3p	5′-CAC​ACT​TGA​AAC​ATA​CAC​GGG​A -3′
miR-539	5′-CTG​CGA​GAG​GAG​AAA​TTA​TCC -3′
miR-490	5′-AAG​CGG​ACC​ATG​GAT​CTC​CA -3′
miR-331	5′-AAG​AAT​GCC​CCT​GGG​CCT​AT -3′
miR-296	5′- AGGGCCCCCCCTCAAT -3′
miR-27a	5′- AAT​CCG​GAT​TCA​CAG​TGG​CTA​A-3′
miR-125b-1	5′- AAT​GCA​CAC​GGG​TTA​GGC​TC-3′
miR-3588	5′- GAG​CAA​ACA​ACC​CAT​GAC​GAA​ACG -3′
miR-3570	5′- GGC​TCT​GAA​AGG​CAA​GAC​AAG-3′
miR-3543	5′- GAT​GAC​CAC​GCC​CTC​CAA​ACA​G-3′
miR-341	5′- TCC​TGG​TGG​TGG​TTC​TGT​CGA​TC -3′
miR-3576	5′- CTC​TCT​TGG​CAG​CCT​TCC​TGA​TTT​C -3′
U6	5′- GCT​TCG​GCA​GCA​CAT​ATA​CTA​AAA​T-3′
FosB-F	5′- GTG​AGA​GAT​TTG​CCA​GGG​TC-3′
FosB-R	5′- AGA​GAG​AAG​CCG​TCA​GGT​TG-3′
LIMK1-F	5′- GCC​TTC​GCT​CTT​GCT​TCG​T-3′
LIMK1-R	5′- TTC​CCT​CTG​CCT​AGC​CTC​TGT-3′
MAP2K4-F	5′- CAA​CAC​TGG​GAT​TTC​ACT​GCA​G-3′
MAP2K4-R	5′- TAT​GGG​CAA​TCA​CTA​CTC​CGC-3′
GAPDH-F	5′- ATT​GTC​AGC​AAT​GCA​TCC​TG-3′
GAPDH-R	5′- ATG​GGA​CTG​TGG​TCA​TGA​GCC-3′

### Transfection of miR-27a-3p inhibitor into PC12 cells

PC12 cells were divided into four groups: negative control (NC), NC + KET, inhibitor + KET, and inhibitor + KET + l-THP. Cells were seeded at a density of 1.25 × 105 cells/well in 12-well plates and incubated with 10 nmol/L NC/miR-27a-3p inhibitor at 50% confluency. After 24 h of culture, the cells were exposed to KET for 48 h. In the l-THP-treated group, the cells were pre-cultivated with l-THP for 15 min before incubation with KET.

### Statistical analysis

Data are presented as mean ± SEM. CPP scores = (post-test time − pre-test time in drug-paired chamber). The importance of the differences among groups was assessed using one-way analysis of variance (ANOVA) through SPSS (version 27.0; SPSS, Inc., Chicago, IL, United States). When required, a *post hoc* test adjusted with Bonferroni correction was utilized. When comparing only two groups, the Student’s t-test was applied. A *p*-value of less than 0.05 was considered to indicate statistical significance.

## Results

### l-THP attenuates KET-induced CPP

As shown in Figures 1C–E, KET induced a significant preference for the drug-associated environment, with a mean conditioning score of 149.5 ± 36.1 s. Pretreatment with l-THP significantly inhibited this effect, reducing the mean conditioning score to 29.7 ± 14.1 s (F (2, 15) = 6.40, *p =* 0.012). Thus, we concluded that the KET-induced CPP model was successfully established. Treatment with l-THP notably reduced the duration that rats spent in the white box, in contrast to the KET group. We can find that l-THP significantly attenuates KET-induced CPP in rats. As depicted in Figures 1D,E, there was no significant difference in the total distance travelled or the number of shuttles (*p* > 0.05).

### l-THP alleviates pathological injury in the hippocampus of rats with KET-induced CPP

In contrast to the Control group, pyramidal cells in the hippocampal CA1 region of KET group rats had a loose arrangement, disordered hierarchy, irregular shape, more cell deletion, concentrated and deeply stained nuclei, and cytoplasmic shrinkage. However, in contrast to the KET group, the pyramidal cells in the hippocampal CA1 region of the K + T group were arranged neatly and had a clear structure (Figure 1G). As illustrated in Figure 1H, compared to the Control group, the neurons of the KET group were sparse, disarrayed, and severely necrotic, exhibiting shrunken nuclei and a marked reduction in Nissl bodies, indicative of extensive cellular damage. However, compared to the KET group, neuronal necrosis in the K + T group was less pronounced, with improved cellular morphology, tighter arrangement, and a notable increase in Nissl bodies. These findings indicate that l-THP treatment significantly alleviates hippocampal injury in rats with KET-induced CPP.

### l-THP regulates hippocampal BDNF-TrkB and downstream pathway expression in KET-induced CPP

**FIGURE 2:**
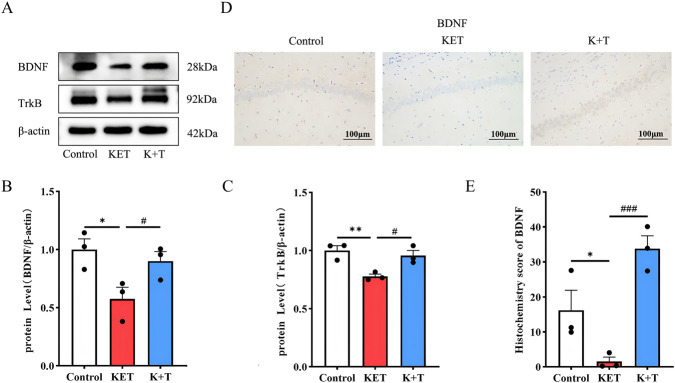
Effect of l-THP on hippocampal BDNF and TrkB in KET-induced CPP. **(A)** Representative immunoblots. Graphic representation of the relative expression of BDNF to β-actin **(B)** and TrkB to β-actin **(C)**. **(D)** Immunohistochemical staining for BDNF expression. Figures were magnified by ×200. **(E)** Comparison of the percentage of BDNF-positive cells. Data are presented as the mean ± SEM (*n* = 3). *p < 0.05, **p < 0.01 KET group vs. Control group; ^#^ *p* < 0.05, ^##^ *p* < 0.01, ^###^ *p* < 0.001K + T group vs. KET group.

To confirm the impact of l-THP on hippocampal BDNF-TrkB expression in KET-induced CPP, BDNF and TrKB levels were examined utilizing Western blotting and immunohistochemistry. In contrast to the Control group, the KET group exhibited a substantial decrease in the protein expression of BDNF and TrkB (*p =* 0.039 and *p* = 0.008, respectively). Nevertheless, when contrasted with the KET group, l-THP markedly enhanced the expression levels of BDNF and TrkB (*p =* 0.043 and *p* = 0.031, Figures 2A–C). Moreover, the proportion of BDNF-positive cells in the KET group was markedly reduced in contrast to the Control group. The group that received l-THP treatment showed a greater percentage of BDNF-positive cells than the KET group (F (2, 6) = 16.57, *p* < 0.001, Figures 2D,E).

**FIGURE 3:**
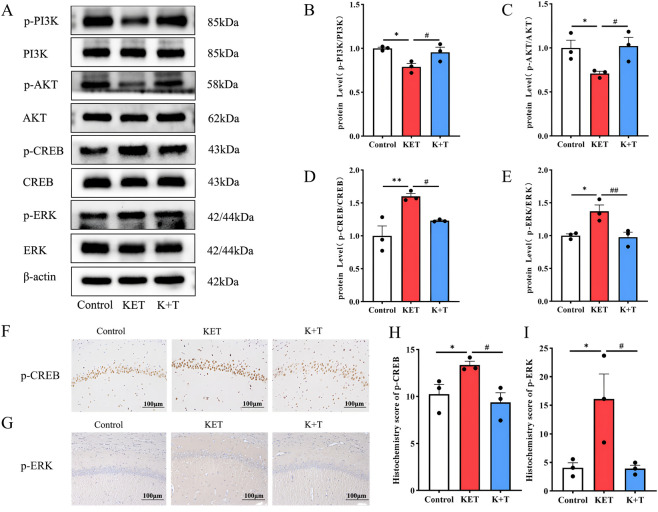
l-THP modulates the BDNF-TrkB downstream signaling pathway in KET-induced CPP. **(A)** Representative immunoblots. Graphic representation of the relative expression of p-PI3K to PI3K **(B)**, p-AKT to AKT **(C)**, p-CREB to CREB **(D)**, and p-ERK to ERK **(E)**. Immunohistochemical staining for p-CREB expression **(F)** and p-ERK expression **(G)**. Comparison of the percentage of p-CREB positive cells **(H)** and p-ERK positive cells **(I)**. Figures were magnified by ×200. Data are presented as the mean ± SEM (*n* = 3). *p < 0.05, **p < 0.01, KET group vs. Control group; ^#^ *p* < 0.05, ^##^ *p* < 0.01, K + T group vs. KET group.

To investigate additional changes related to BDNF-TrkB signalling following l-THP treatment, the downstream pathway was identified. (Figures 3A–E). In contrast to the Control group, the KET group showed a decrease in the expression levels of p-PI3K and p-AKT (*p =* 0.024 and *p* = 0.048, respectively). Nevertheless, treatment with l-THP markedly restored the alterations in p-PI3K and p-AKT levels observed in the K + T group (*p =* 0.038 and *p* = 0.046, respectively). Moreover, the KET group exhibited elevated levels of p-ERK and p-CREB compared to the Control group (, *p =* 0.024 and *p* = 0.008, respectively), whereas treatment with l-THP decreased the levels of p-ERK and p-CREB compared to those in the KET group (*p =* 0.019 and *p* = 0.033, respectively). In the KET group, the proportion of cells positive for p-CREB and p-ERK was markedly greater compared to the Control group (*p =* 0.038 and *p* = 0.039, respectively). l-THP decreased the percentage of p-CREB and p-ERK positive cells in contrast to the KET group (*p =* 0.04 and *p* = 0.037, Figures 3F–I).

### l-THP changes miRNA hippocampal expression profiles in rats with KET-induced CPP

**FIGURE 4:**
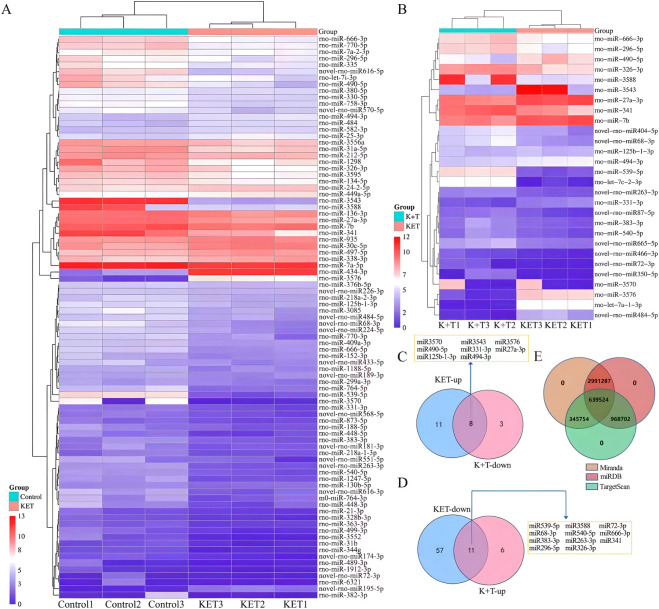
**(A, B)** Clustering analysis of differentially expressed miRNAs between different groups. The color bar of A is from 0 to 13. The color bar of B is from 0 to 12. They represent the expression levels of different miRNAs. **(C)** Eight miRNAs were upregulated in the KET group and downregulated in the K + T group. **(D)** Eleven miRNAs were downregulated in the KET group and upregulated in the K + T group. **(E)** Venn diagram of prediction of the target genes by Miranda, TargetScan, and miRDB database.

**FIGURE 5:**
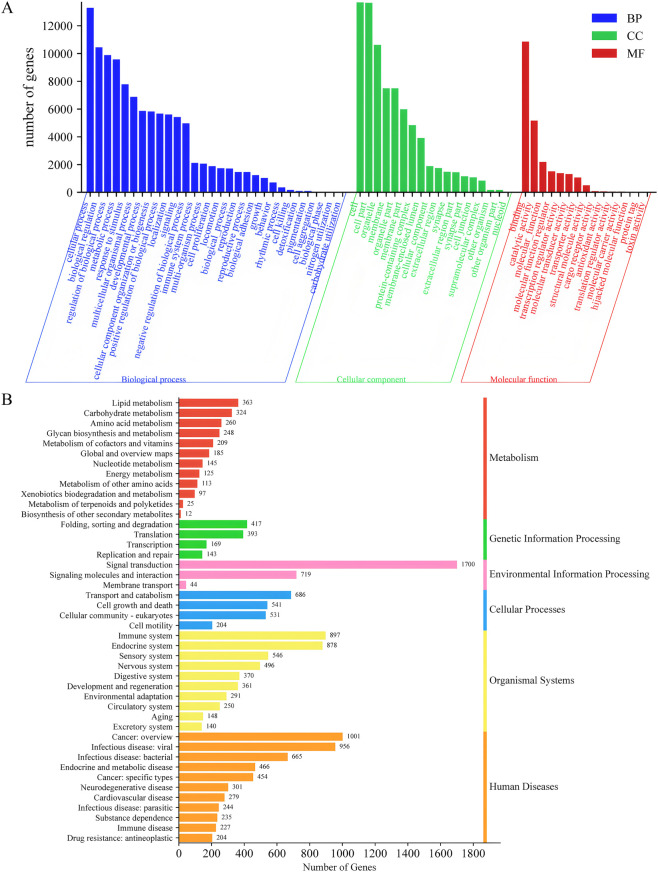
**(A)** A gene ontology (GO) analysis. **(B)** Kyoto Encyclopedia of genes and genomes (KEGG) analysis.

**FIGURE 6:**
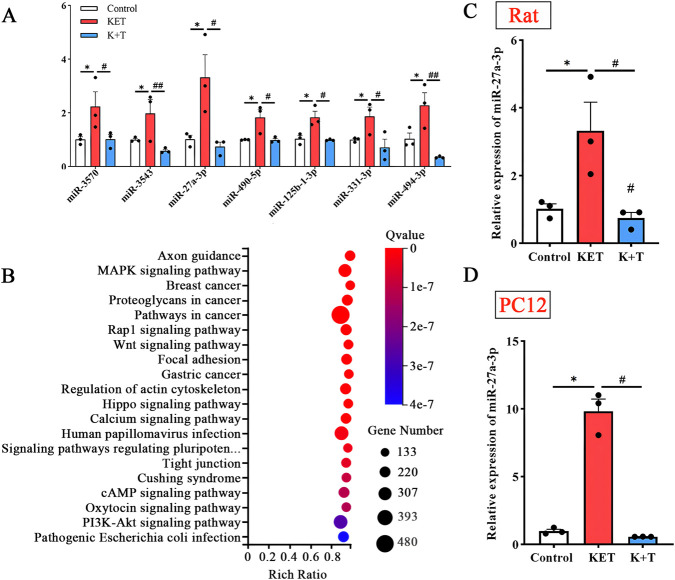
**(A)** Validation of differential miRNAs through RT-PCR. **(B)** KEGG pathway enrichment bubble chart. **(C)** Compared with the KET group, the expression of miR-27a-3p was significantly reduced in rat hippocampus in the K + T group. **(D)** Compared with the KET group, the expression of miR-27a-3p was markedly reduced in PC12 cells in the K + T group. Values are shown as mean ± SEM, *n* = 3 per group. *p < 0.05, KET group vs. Control group; ^#^ *p* < 0.05, ^##^ *p* < 0.01, K + T group vs. KET group.

To elucidate the molecular mechanism by which l-THP modulates KET-induced CPP, next-generation high-throughput sequencing was employed to profile hippocampal miRNA expression. Comparative analysis revealed 87 miRNAs with significantly altered expression levels in the KET group relative to Controls (Figure 4A), while l-THP treatment induced differential expression of 28 miRNAs compared to the KET group (Figure 4B; Table 2). As shown in Figure 4E, a total of 639,524 target genes were predicted based on the differentially expressed miRNAs between the Control and KET groups, as well as between the KET and K + T group using Miranda, TargetScan, and miRDB databases. Then, the 639,524 target genes were subjected to GO and KEGG pathway analyses. GO annotation revealed enrichment of genes involved in biological processes (BP), cellular components (CC), and molecular functions (MF) (Figure 5A), including cellular functions, biological control, and metabolic pathways—all implicated in drug addiction (Zhang et al., 2020). KEGG analysis further demonstrated involvement in cellular processes, environmental information processing, and organismal systems (Figure 5B), with specific pathways such as lipid metabolism, signal transduction, and substance reward effect linked to morphine-induced CPP (Zhang et al., 2020). Notably, KEGG pathway enrichment analysis highlighted the MAPK signaling pathway as a key mediator of l-THP’s intervention in KET addiction (Figure 6B), suggesting a potential molecular target for therapeutic modulation.

**TABLE 2:** Different hippocampal^1^ miRNAs in the KET and K + T groups.

miRNA	Alignments	Sequence	Fold change (THP/K)	*P* value (THP/K)
Upregulation				
novel-rno-miR263-3p	chr11:58661267-58661326	UCU​GAA​GCU​UGC​UUA​CCU​CC	3.031	1.59E-6
novel-rno-miR350-5p	chrX:33516113-33516172	UGG​GGG​CAG​GCC​GGA​UCU​AGU	59.714	4.25E-5
novel-rno-miR404-5p	chr1:126523307-126523356	GCGCGGGCCGGAGCGGGG	4.258	5E-8
novel-rno-miR466-3p	chr14:7230302-7230346	GUGGGUGGUGGUGCAUGG	5.098	9.27E-4
novel-rno-miR665-5p	chr1:174495293-174495366	GCG​GGC​GGG​GCC​GGG​GGU​G	3.555	9E-8
novel-rno-miR68-3p	chr11:36383598-36383663	UCU​GCU​GAC​UGC​CUA​UGG​GCU	4.500	2E-8
novel-rno-miR72-3p	chr4:9191348-9191405	UGU​UCU​GUG​CUC​UGU​CAC​UCA​GG	22.162	9.23E-4
novel-rno-miR87-5p	chr8:53864333-53864404	GGGCGGGGCCGGGGGUGG	3.249	5.71E-4
rno-let-7c-2-3p	chr7:126590212-126590306	CUA​UAC​AAU​CUA​CUG​UCU​UUC​C	227.544	2.0E-12
rno-miR-296-5p	chr3:172357490-172357567	AGG​GCC​CCC​CCU​CAA​UCC​UGU	2.585	8.61E-6
rno-miR-326-3p	chr1:164518401-164518495	CCU​CUG​GGC​CCU​UCC​UCC​AGU	2.514	1.36E-4
rno-miR-341	chr6:133733240-133733335	UCG​GUC​GAU​CGG​UCG​GUC​GGU	2.713	3.43E-4
rno-miR-3588	chr11:16097337-16097442	UCA​CAA​GUU​AGG​GUC​UCA​GGG​A	40.786	2.69E-5
rno-miR-383-3p	chr16:57519675-57519748	CCA​CAG​CAC​UGC​CUG​GUC​AGA	3.138	5.23E-4
rno-miR-539-5p	chr6:133877832-133877907	GGA​GAA​AUU​AUC​CUU​GGU​GUG​U	62.683	1.89E-109
rno-miR-540-5p	chr6:133706202-133706285	CAA​GGG​UCA​CCC​UCU​GAC​UCU​GU	3.182	5.67E-4
rno-miR-666-3p	chr6:133866523-133866621	GGC​UGC​AGC​GUG​AUC​GCC​UGC​UC	2.168	1.20E-4
rno-let-7a-1-3p	chr17:16417853-16417946	CUA​UAC​AAU​CUA​CUG​UCU​UUC​C	2.300	3.7E-30
Downregulation				
rno-miR-27a-3p	chr19:25318736-25318822	UUC​ACA​GUG​GCU​AAG​UUC​CGC	0.398	4E-8
rno-miR-3576	chr6:133877812-133877926	ACA​CAC​CAA​GGA​UAA​UUU​CUC​C	0.007	4.6E-43
rno-miR-494-3p	chr6:133864370-133864452	UGA​AAC​AUA​CAC​GGG​AAA​CCU​CU	0.490	6.75E-6
rno-miR-3570	chr13:54952723-54952839	GGU​ACA​AUC​AAC​GGU​CGA​UGG​U	0.420	4.44E-4
rno-miR-3543	chr6:133716215-133716329	CAG​GAG​UCG​AGU​GAU​GGU​UCA​AA	0.005	3E-7
rno-miR-490-5p	chr4:63342943-63343026	CCA​UGG​AUC​UCC​AGG​UGG​GU	0.232	3.66E-6
rno-miR-125b-1-3p	chr8:45798260-45798346	ACG​GGU​UAG​GCU​CUU​GGG​AGC​U	0.466	1.91E-4
novel-rno-miR484-5p	chr11:58661267-58661326	CUA​AGG​CAG​GCA​GAC​UUC​AGU	0.021	7.8E-21
rno-miR-331-3p	chr7:34881095-34881190	GCC​CCU​GGG​CCU​AUC​CUA​GAA	0.295	1.02E-5
rno-miR-7b	chr9:10812432-10812541	UGG​AAG​ACU​UGU​GAU​UUU​GUU​GU	0.449	3.93E-6

1 Twenty-eight miRNAs, selected from the next-generation miRNA, sequencing data are presented; 18 upregulated and 10 downregulated miRNA, profiles were divided, and the genomic locus of the chromosome and the sequence of the miRNAs, are listed. Additionally, the accessions and fold-changes for each miRNA, are shown.

### l-THP reverse the increased expression of miR-27a-3p in rat hippocampus and PC12 cells

Further screening indicated that 19 miRNAs were differentially expressed miRNAs between KET and K + T groups (Figures 4C,D; Table 3). Based on their significance and potential functional predictions, we selected 12 miRNAs from them for RT-qPCR verification. In contrast to the Control group, seven were significantly upregulated in the KET group (Figure 6A). However, l-THP reversed the upregulation of these seven miRNAs (Figure 6A). Considering the dual validation outcomes of RT-qPCR and next-generation high-throughput sequencing, and the important functions of miR-27a-3p in brain neurological processes, we focused on miR-27a-3p miRNA for subsequent functional analysis. The expression of miR-27a-3p in rat hippocampus and PC12 cells was verified by RT-qPCR (Figures 6C,D). The levels of miR-27a-3p showed a significant increase in the KET group, and downregulated after treatment with l-THP in both rat hippocampus and PC12 cells (F (2, 6) = 7.89, p = 0.041 and F (2, 6) = 12.93, p = 0.022, respectively). Taken together, these results indicate that l-THP attenuates the upregulated expression of miR-27a-3p in the hippocampus of KET-dependent rats and in PC12 cells. These findings suggest that the miR-27a-3p/MAP2K4 axis may contribute to the attenuating effect of l-THP on KET reward.

**TABLE 3:** Specific information on 19 miRNAs in the Venn diagram depicted in Figures 4C,D.

miRNA	Fold change (THP/K)	*p*-value
rno-miR-3570	0.420	4.44E-04
rno-miR-3543	0.005	3.00E-07
rno-miR-3576	0.007	4.60E-43
rno-miR-539-5p	62.683	1.89E-109
rno-miR-3588	40.786	2.69E-05
novel-rno-miR72-3p	22.162	9.23E-04
novel-rno-miR68-3p	4.500	2.00E-08
rno-miR-490-5p	0.232	3.66E-06
rno-miR-331-3p	0.295	1.02E-05
rno-miR-540-5p	3.182	5.67E-04
rno-miR-666-3p	2.168	1.20E-04
rno-miR-383-3p	3.138	5.23E-04
novel-rno-miR263-3p	3.031	1.59E-06
rno-miR-341	2.713	3.43E-04
rno-miR-296-5p	2.585	8.61E-06
rno-miR-27a-3p	0.398	4.00E-08
rno-miR-326-3p	2.514	1.36E-04
rno-miR-125b-1-3p	0.466	1.91E-04
rno-miR-494-3p	0.490	6.75E-06

**FIGURE 7:**
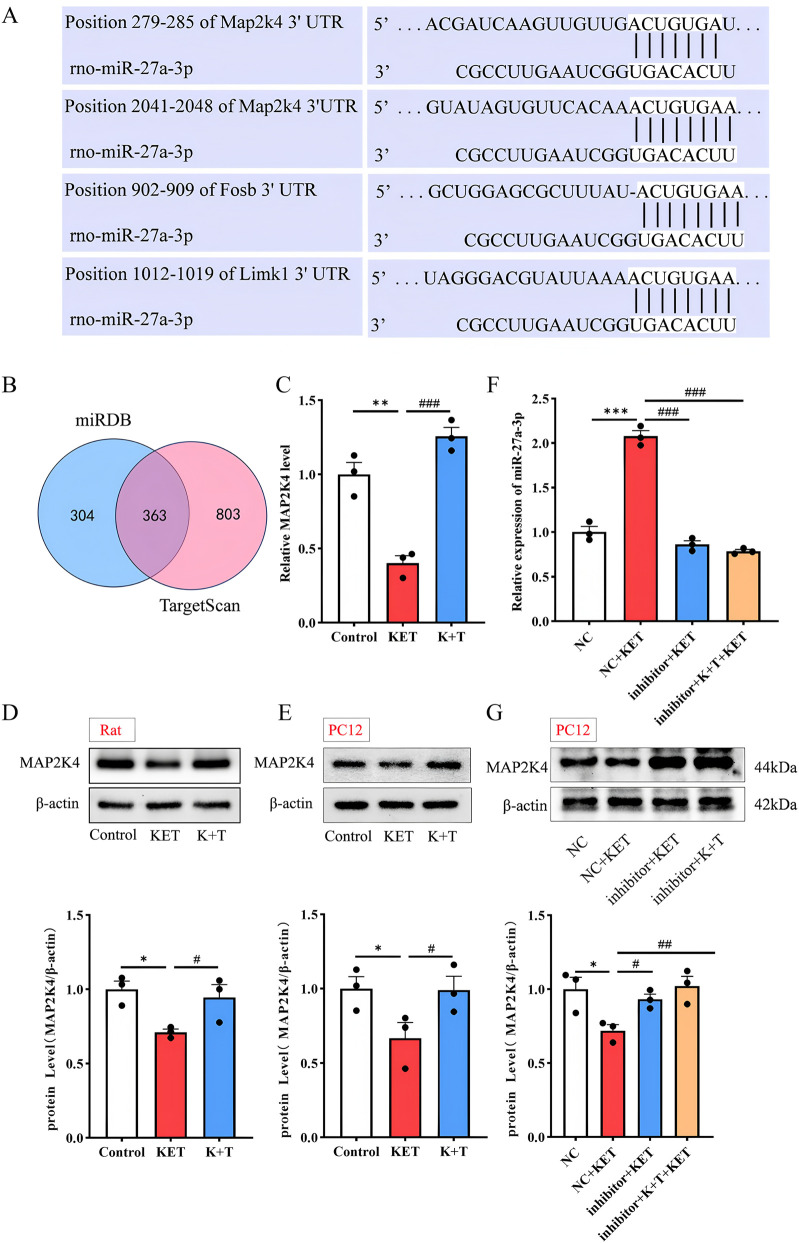
**(A)** Limk1, MAPK2K4, and Fosb gene sequence alignment of miR-27a-3p to 3′-UTR. **(B)** miR-27a-3p target genes were determined using three target gene prediction databases (TargetScan, miRDB, and miRanda). MiRanda could not predict the target genes related to miR-27a-3p. **(C)** The expression of MAP2K4 mRNA in hippocampal tissue was detected by RT-qPCR. **p < 0.01, KET group vs. control group, ^###^ *p* < 0.001, K + T group vs. KET group. **(D)** Compared to the KET group, MAP2K4 protein expression was significantly increased in rat hippocampus in the K + T group. **(E)** Compared to the KET group, MAP2K4 protein expression was markedly increased in PC12 cells in the K + T group. *p < 0.05, KET group vs. control group, ^#^ *p* < 0.05, K + T group vs. KET group. **(F)** After transfection of NC and miR-27a-3p inhibitors into PC12 cells, qRT-PCR was used to quantitate miR-27a-3p expression in the cells. ***p < 0.001, NC + KET group vs. NC group, ^###^ *p* < 0.001, inhibitor + KET group vs. NC + KET group and inhibitor + K + T group vs. NC + KET group. **(G)** Western blotting was used to quantify the protein expression levels of MAP2K4. Values are shown as mean ± SEM, *n* = 3 per group. *p < 0.05, NC + KET group vs. NC group. ^#^ *p* < 0.05, ^##^ *p* < 0.01, inhibitor + KET group vs. NC + KET group and inhibitor + K + T group vs. NC + KET group.

To investigate the regulatory mechanisms underlying l-THP-induced suppression of miR-27a-3p in KET reward effect, the target genes of miR-27a-3p were determined. Three leading prediction databases, TargetScan, miRDB, and miRanda were used for target gene prediction. MiRanda could not predict the target genes related to miR-27a-3p. Collectively, TargetScan and miRDB databases identified 363 probable target genes (Figure 7B). Literature analysis revealed that MAP2K4, FosB, and LIMK1 are closely associated with neurological diseases and contain highly conserved binding sites for miR-27a-3p (Figure 7A). We evaluated the mRNA levels of MAP2K4, LIMK1, and FosB by RT-qPCR. The results indicated that KET significantly decreased MAP2K4 mRNA expression (*p =* 0.001) while l-THP treatment reversed this decrease in rat hippocampus (*p* < 0.001, Figure 7C). There was no significant difference in the gene expression of LIMK1 and FosB between KET and K + T group. We then quantified the MAP2K4 protein levels by Western blotting (Figures 7D,E). KET significantly decreased MAP2K4 protein expression (*p =* 0.034 and *p* = 0.041, respectively), while treatment with l-THP significantly increased MAP2K4 protein expression in both rat hippocampus and PC12 cells (*p =* 0.028 and *p* = 0.042, respectively, Figures 7D,E). After transfection of the NC and the miR-27a-3p inhibitors into PC12 cells, qRT-PCR was performed. As shown in Figure 7F, the expression of miR-27a-3p (F (3, 8) = 152.00, *p <* 0.001) was markedly reduced in PC12 cells in the inhibitor + KET group and inhibitor + K + T group compared to that in the NC + KET group. Taken together, these results suggest that PC12 cells were successfully transfected with the miR-27a-3p inhibitor.

Furthermore, we studied MAP2K4 protein levels after miR-27a-3p suppression *in vitro*. As shown in Figure 7G, suppression of miR-27a-3p by the miR-27a-3p inhibitor and treatment with l-THP induced a significant increase in MAP2K4 protein expression (F (3, 8) = 5.52, *p =* 0.027 and *p* = 0.008). Collectively, these findings suggest that l-THP may modulate KET reward effect by regulating the miR-27a-3p/MAP2K4 axis.

## Discussion

As a popular bioactive component in *Corydalis ambigua* and *Stephania tetrandra*, l-THP has been extensively studied for its potent pharmacological anti-addictive and neuroprotective properties (Du et al., 2022). In previous studies, we showed that l-THP reduced the KET-induced CPP in rats (Du et al., 2017), and that the underlying principle could be closely associated with the regulation of central neurotransmitters and the expression of p-ERK and p-CREB in the rat brain. In this study, we successfully developed a KET-induced CPP rat model and found that l-THP can suppress KET-induced CPP. In addition, our previous experimental results indicated that l-THP did not lead to changes in behavior.

The mesolimbic DA system has a significant influence on the development of drug addiction. Reports have demonstrated that BDNF has the potential to modulate DA release and influence drug-addictive behavior via TrkB (Liang et al., 2011). To confirm the impacts of l-THP on hippocampal BDNF and TrkB expression in KET-induced CPP, we evaluated their levels by Western blotting and immunohistochemistry. Our findings showed that the levels of BDNF and TrkB were reduced in the KET group when compared to the control group. However, treatment with l-THP led to an increase in the levels of BDNF and TrkB in comparison to the KET group. These findings align with the results from a prior study (Zhang et al., 2021). To further investigate the changes after l-THP treatment, we explored the downstream pathways of BDNF-TrkB. Previous studies have indicated that the activation of BDNF-TrkB leads to two primary pathways: (1) the PI3K-AKT pathway, which results in the stimulation of PI3K and AKT and controls cellular viability (Kaplan and Miller, 1997), and (2) the MAP-ERK pathway. In our previous research, it was discovered that chronic KET enhanced the activity of ERK, specifically within the hippocampus (Du et al., 2017). In this research, it was discovered that l-THP increased the levels of p-PI3K and p-AKT, which were reduced in KET-induced CPP. Simultaneously, l-THP decreased the levels of p-ERK and p-CREB, which increased in KET-induced CPP. These results are consistent with previous reports (Li et al., 2018; Zhang et al., 2021). CREB is essential in the process of perceiving seeking drugs and experiencing rewarding effects (McPherson and Lawrence, 2007). The findings demonstrate that l-THP treatment for KET-induced CPP is associated with the BDNF-TrkB signalling pathway and its subsequent downstream mechanisms.

Beyond classical neurotrophic signaling, ketamine exerts its effects through multiple alternative and compensatory pathways. As a non-competitive NMDA receptor antagonist, ketamine reduces GABAergic inhibition to increase glutamate release, activating AMPA receptors and triggering downstream mTOR signaling—this promotes synaptic protein synthesis and dendritic spine formation independently of neurotrophins (Abdallah et al., 2020; Antos et al., 2024). When combined with cerebrolysin, ketamine further benefits from the upregulation of the CREB/PGC-1α pathway, which improves mitochondrial function by reducing ROS production and increasing ATP levels, thereby enhancing neuronal survival and cognitive/emotional behaviors (Hosseini et al., 2025). Additionally, rapamycin prolongs ketamine’s antidepressant effects via anti-inflammatory actions and autophagy enhancement, protecting newly formed synapses from inflammatory damage without altering ketamine’s blood levels or psychotomimetic effects (Abdallah et al., 2020; Duman, 2002). Ketamine’s metabolites (e.g., (2R,6R)-hydroxynorketamine) also play a role, directly interacting with TrkB receptors to restore neuronal plasticity without relying on increased BDNF levels (Antos et al., 2024). These pathways collectively compensate for reduced receptor/neurotrophin levels, sustaining neuroprotective and behavioral effects.

In summary, ketamine’s therapeutic effects involve multiple alternative and compensatory cascades—including AMPA-mTOR activation, CREB/PGC-1α-mediated mitochondrial regulation, and metabolite-driven TrkB modulation—that collectively account for the observed inconsistency between downstream neurotrophic signaling and receptor/neurotrophin level reductions. These pathways independently or synergistically sustain neuronal function and synaptic plasticity, highlighting the complexity of ketamine’s mechanism of action beyond classical neurotrophic signaling.

In this present research, we explored the role of miRNAs in controlling KET reward effect using l-THP because they are more deeply involved in gene regulatory processes in the brain, especially their general role in hippocampal synapses (Schratt, 2009). The hippocampus is a crucial component of the limbic system and is involved in the acquisition and control of knowledge, retention processes, emotional responses, and conditioning. Therefore, we chose the hippocampus as the region of focus. Previous literature indicated that miRNAs are essential in playing a key role in the reward effect of addictive substances (Smith and Kenny, 2018). To this end, next-generation high-throughput sequencing of the hippocampus was performed. Nineteen miRNAs were differentially expressed among the Control, KET, and K + T groups. GO and KEGG analyses indicated that these miRNAs mainly participate in the immune system, lipid metabolism, neurodegenerative disease, signal transduction, membrane transport, substance reward effect, and nervous system, which have been demonstrated to serve as a crucial factor in KET-induced CPP (Zhang et al., 2021). miRNAs altered by l-THP showed putative targets related to various functions of the hippocampus. Among these, the MAPK signalling pathway is related to l-THP-mediated KET-induced CPP (Zhang et al., 2021), which is thought to be involved in neuronal functions. Reports have indicated that the MAPK signalling pathway plays a role in the modulation of synaptic plasticity. This pathway plays a crucial role in facilitating long-term alterations in memory function and addictive characteristics (Kelleher et al., 2004).

MiR-27a-3p has been demonstrated in prior studies to be involved in neurological diseases, such as regulating cerebral ischemia, reperfusion injury (Li et al., 2021; Ren and Zhou, 2023), the permeability of the cerebral endothelial barrier (Xi et al., 2018; Harati et al., 2022), inflammatory responses, and the apoptosis of hippocampal neurons in epilepsy (Lu et al., 2019). In the present study, miR-27a-3p was significantly overexpressed in the KET-reward effect rat hippocampus and its expression was reduced following l-THP treatment. Che et al. (2017) demonstrated that during chronic cerebral perfusion, miR-27a controls the post-transcriptional expression of lysosomal-associated membrane protein 2 (LAMP-2) and plays a crucial role in regulating autophagy (Che et al., 2017). miR-27a-3p levels have been significantly elevated in the hippocampal expression of epileptic rats (Lu et al., 2019). In addition, the level of miR-27a-3p in the brain tissue of patients with epilepsy was found to be higher than that in the control group (Wang et al., 2024). Our results are consistent with the previously mentioned reports. Therefore, miR-27a-3p may play a crucial role in the alteration of synaptic functions in the hippocampus following KET exposure, resulting in KET reward effect.

PC12 cells, which serve as a model for neuronal cells, were widely used for the investigation of neurotransmitter dynamics, ion channel function, and receptor activity, and were employed to simulate addiction *in vitro*. Previous studies demonstrated that PC12 cell addiction is induced by exposure to amphetamines for 48 h (Kantor et al., 2004). Herein, to mimic the duration of cellular addiction, PC12 cells were treated with KET for 48 h. We found that KET significantly increased the expression of miR-27a-3p, whereas l-THP reversed this expression in PC12 cells. Moreover, exposure to KET markedly reduced PC12 cell neurite length, whereas l-THP treatment promoted neurite outgrowth. The inhibitory effect of KET on neurite outgrowth in PC12 cells is probably due to the increased expression of miR-27a-3p. These results indicate that the inhibitory effects of l-THP on KET reward effect are linked to the reduction in miR-27a-3p expression.

We further explored the regulatory mechanism of l-THP-induced suppression of miR-27a-3p in KET-reward effect. Through the prediction of miR-27a-3p targets and qPCR verification, we focused on MAP2K4. MAP2K4 is a member of the MAPK family of proteins. The activated MAPK pathway is related to increased levels of inflammatory mediators and apoptosis in nerve cells (Wang et al., 2019). Previous results of a dual-luciferase reporter assay indicated that miR-27a-3p specifically regulates MAP2K4 at the post-transcriptional level (Wan et al., 2016; Lu et al., 2019; Chen et al., 2020). Further, MAP2K4 was discovered to be a downstream target of miR-27a-3p, the latter suppressing the levels of MAP2K4/JNK and enhancing the protective effects of melatonin, which plays a vital role in safeguarding neural stem cells against the overproduction of reactive oxygen species (Chen et al., 2020). MAP2K4 influences the downstream JNK pathway to activate the MAPK signalling pathway (Chen et al., 2020). The findings of this research indicated that MAP2K4, in contrast to miR-27a-3p, was significantly decreased in the hippocampus of rats with KET-induced CPP and increased after treatment with l-THP. Additionally, inhibition of miR-27a-3p expression using a miR-27a-3p inhibitor in PC12 cells led to increased expression of MAP2K4, mirroring the effects caused by l-THP treatment. Together, these findings demonstrate that l-THP inhibits miR-27a-3p expression, and suggest that MAP2K4 is involved in this regulatory effect on KET reward.

The microRNAs-target gene related to ketamine addiction that have been reported in the current literature mainly include miR-331-5p target Nurr1 (Li et al., 2018), miR-429/BAG5 (Fan et al., 2021), miR-384-5p/GABRB1 (Yang and Long, 2024). The report indicates that miR-27a-3p/MAP2K4 is related to epilepsy (Lu et al., 2019). Our work is the first to (1) identify miR-27a-3p as a key regulator specifically in the context of ketamine-induced conditioned place preference, and (2) We propose a potential mechanistic connection between this pathway and l-THP’s anti-addictive effects, which may involve the targeting of MAP2K4. This provides not only a novel molecular mechanism for l-THP’s action but also a potential therapeutic target (miR-27a-3p/MAP2K4) distinct from conventional opioid or dopamine-focused approach. We incorporated this part of the content into the discussion.

In this experiment, we used only male rats as the research subjects. However, the study has certain limitations. Our future studies should repeat this work in female rat.

## Conclusion

In summary, the results of this research suggest that the suppressive impact of l-THP on KET reward effect is significantly associated with the regulation of hippocampal BDNF/TrkB and its downstream pathways and the miR-27a-3p/MAP2K4 axis. Although further studies on the stereotactic injection of the miR-27a-3p agomir and antagomir in the rat brain are required to elucidate the regulation of the miR-27a-3p/MAP2K4 axis mediated by l-THP in KET reward effect, our research offers new perspectives on the identification of miRNAs as possible specific targets for addressing KET reward effect. Further studies should use l-THP to develop therapeutic agents against KET reward effect.

## Data Availability

The original contributions presented in the study are publicly available. This data can be found in the NCBI repository with the accession number PRJNA1479580.
