# Supplementary material for: L-tetrahydropalmatine attenuates ketamine reward effect via modulating the miR-27a-3p/MAP2K4 axis
Source: Front Genet. 2026 Jun 29;17:1701742. doi: 10.3389/fgene.2026.1701742 (PMC13357822; doi:10.3389/fgene.2026.1701742)

Wetern-Blot picture of rats, Group: Control、KET、K+T

MAP2k4


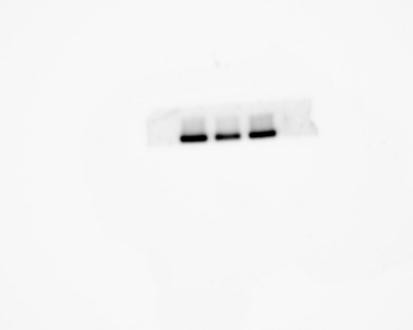

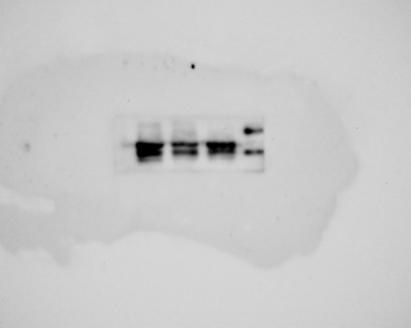

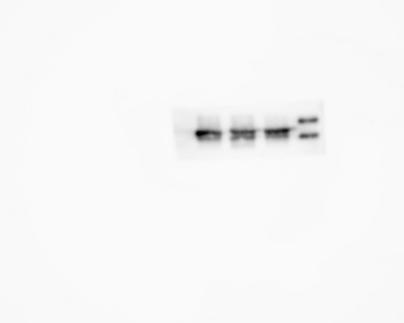


β-actin


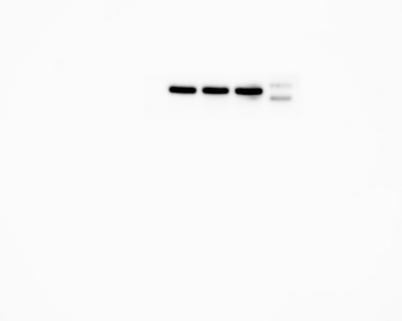

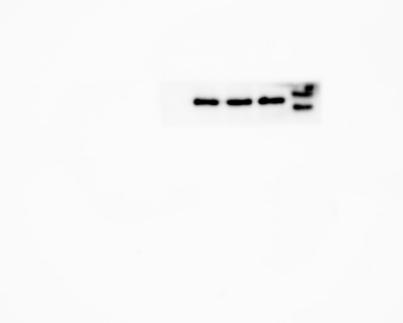

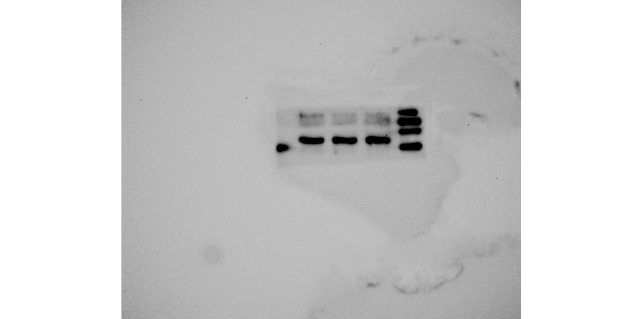


BDNF


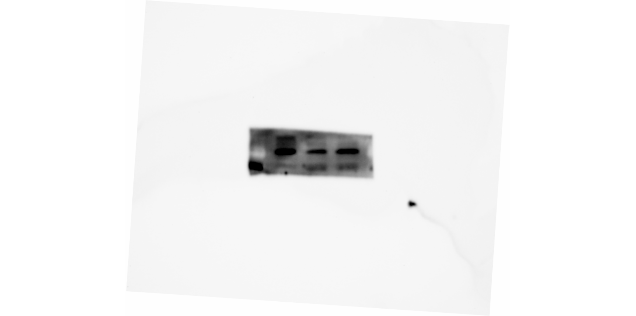

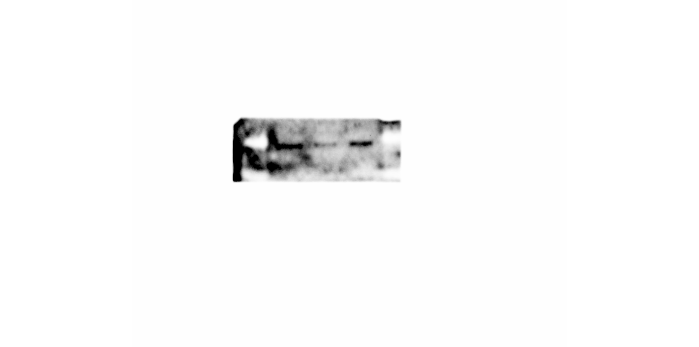

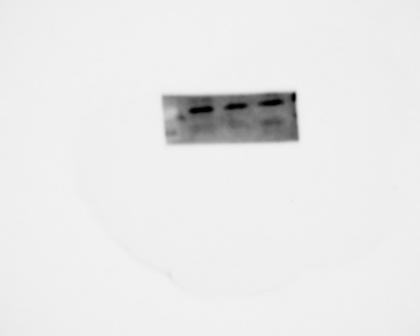


β-actin


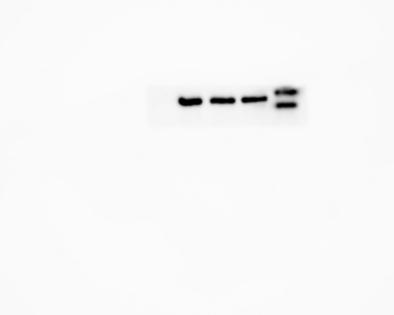

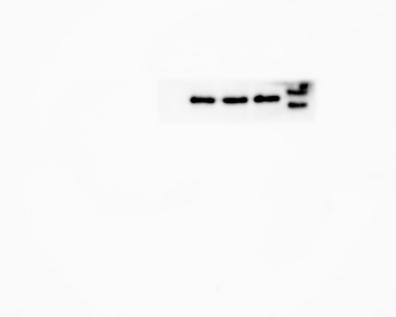

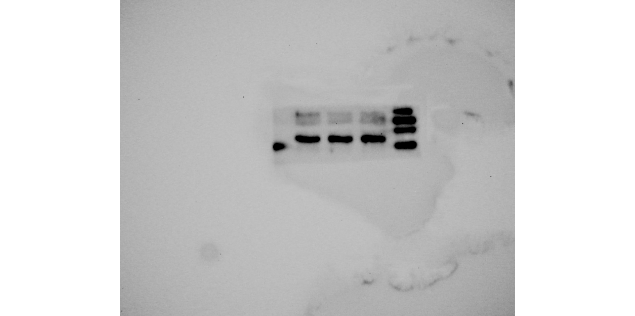


TrkB


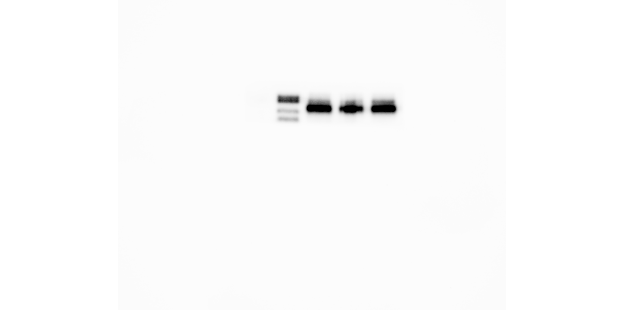

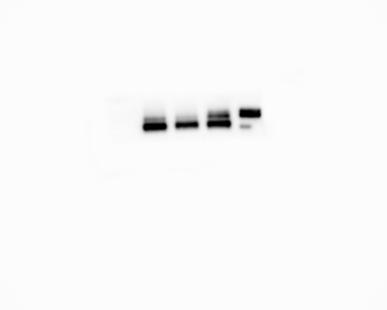

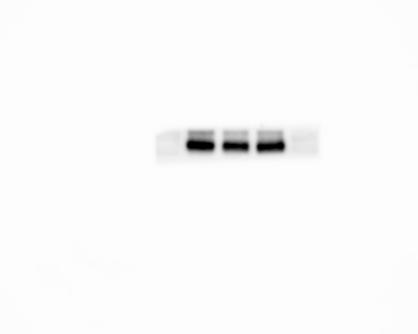


β-actin


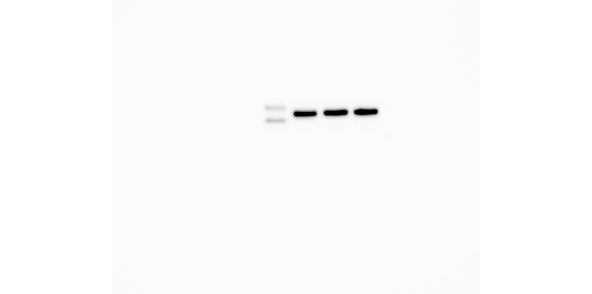

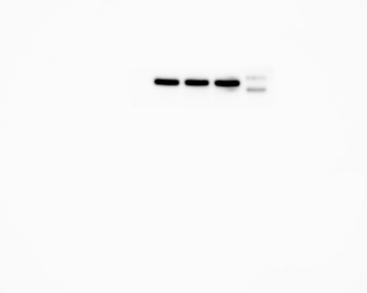

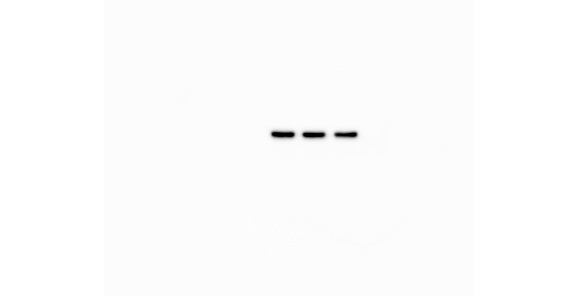


P-PI3K


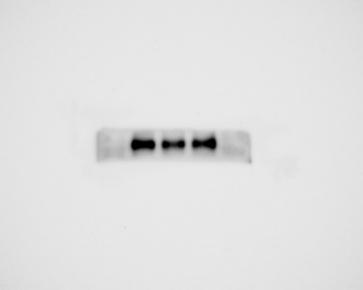

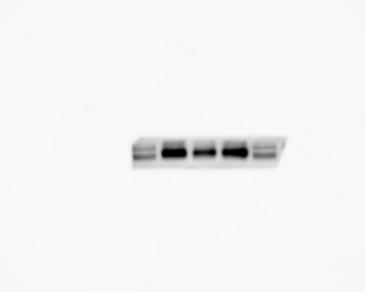

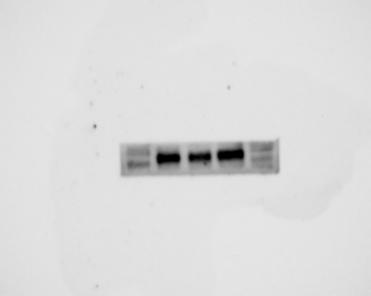


PI3K


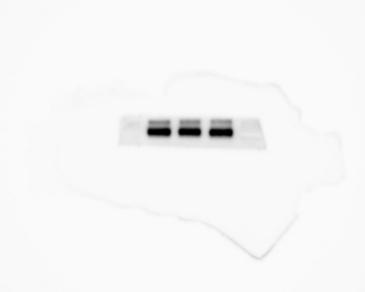

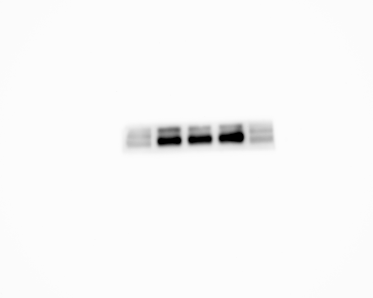

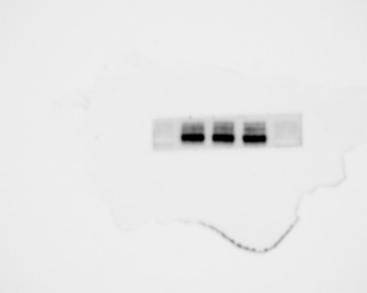


P-AKT


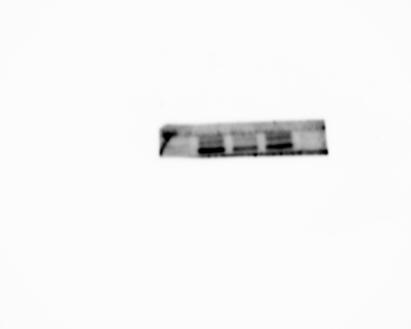

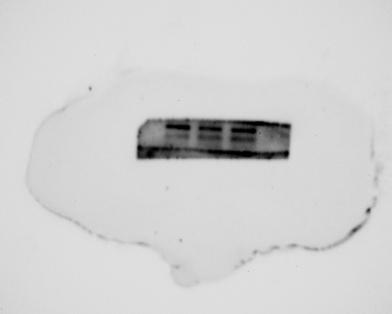

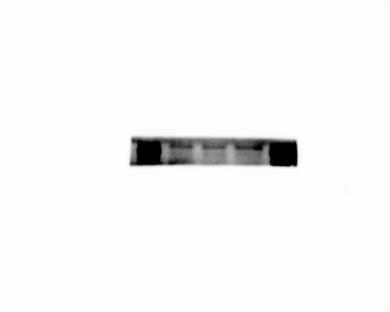


AKT


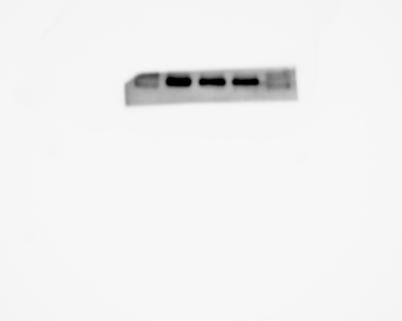

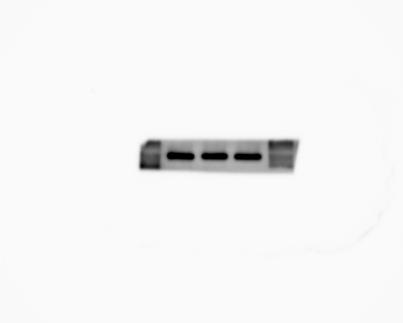

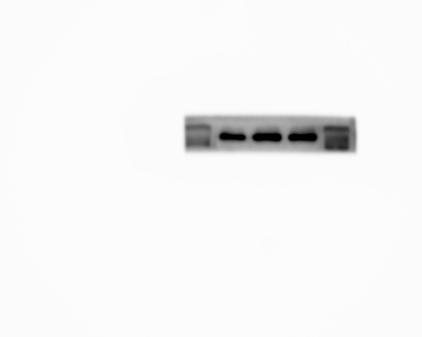


P-ERK


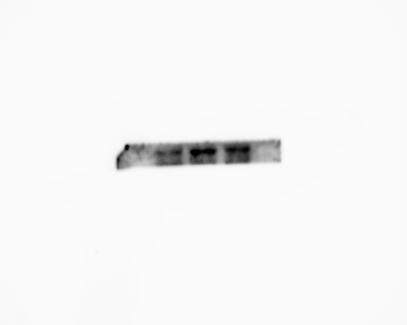

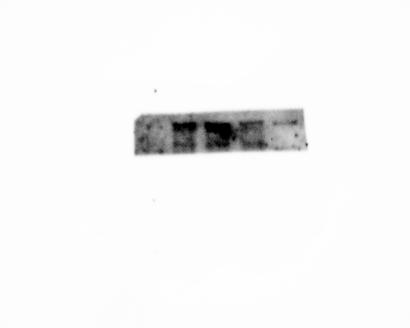

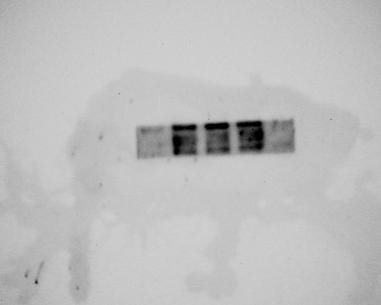


ERK


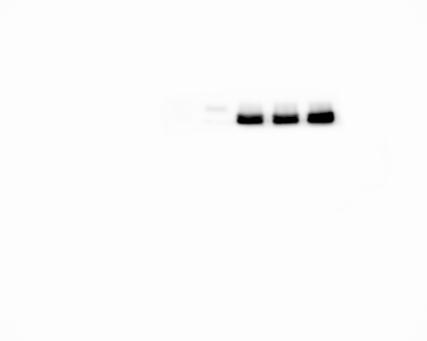

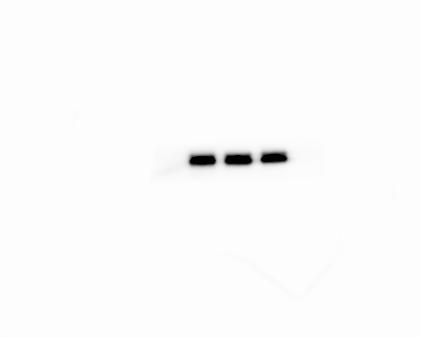

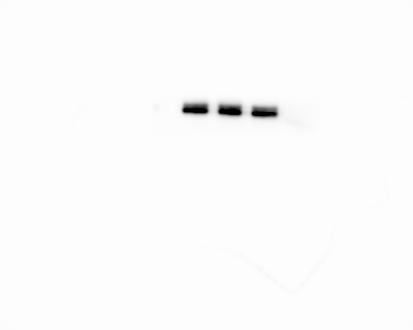


P-CREB


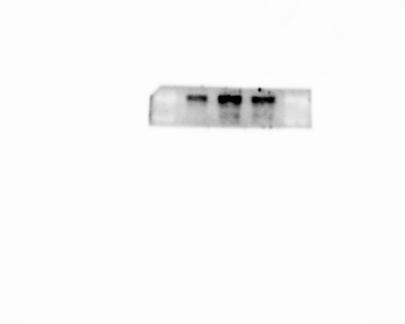

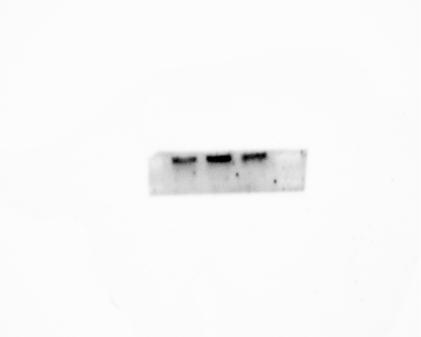

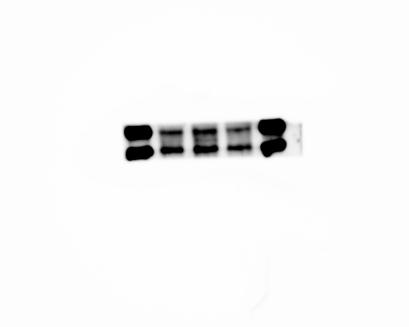


CREB


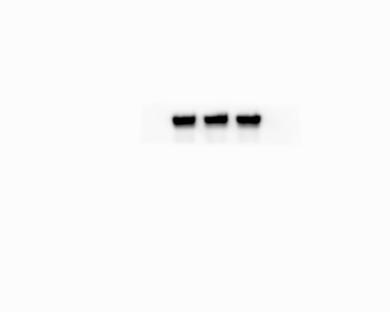

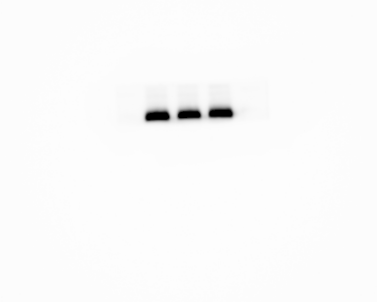

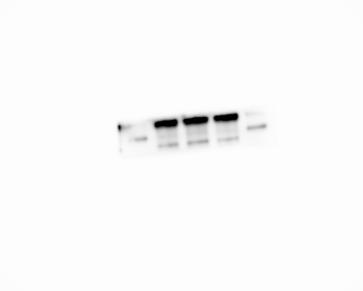


Western-Blot picture of cell

Before transfection, Group: NC、KET、K+T

MAP2K4


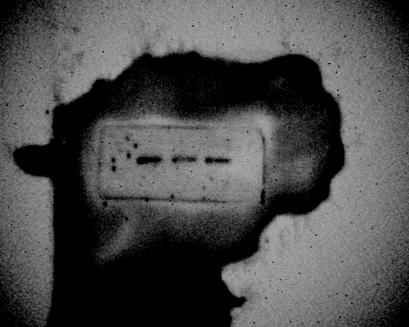

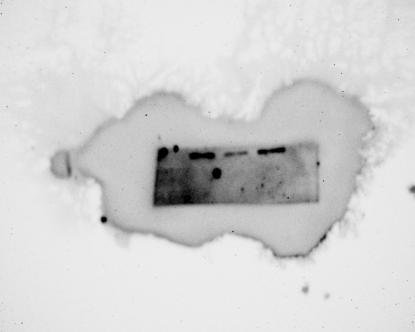

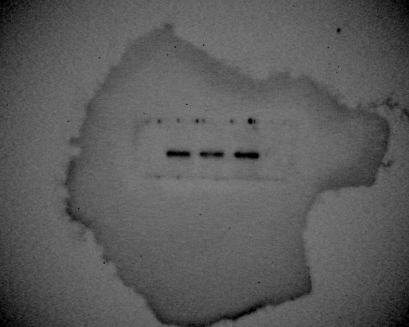


β-actin


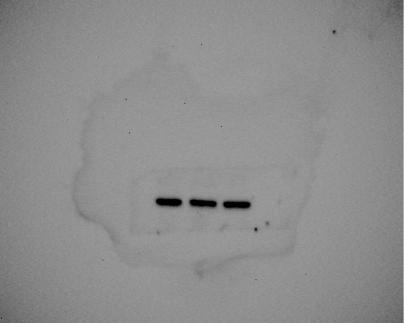

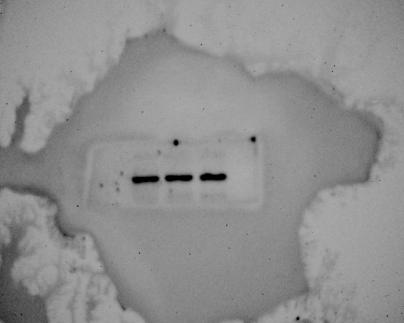

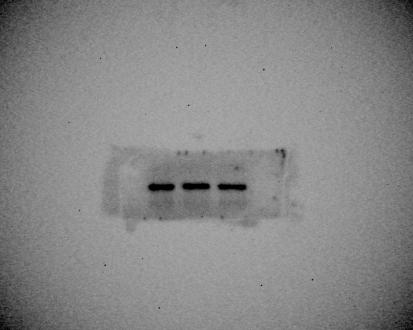


After transfection, Group: NC、KET、inhibitor+KET、inhibitor+K+T

MAP2K4


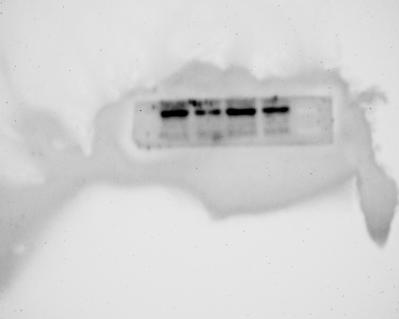

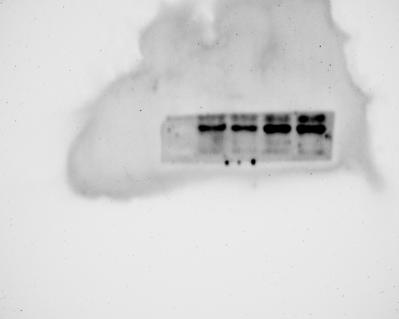

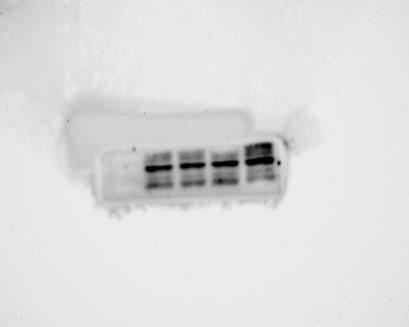


β-actin


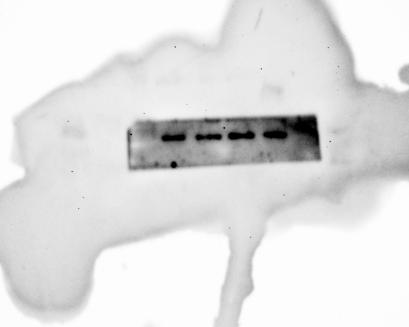

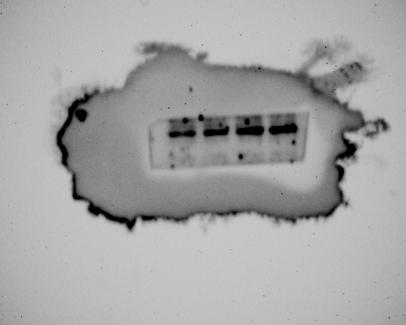

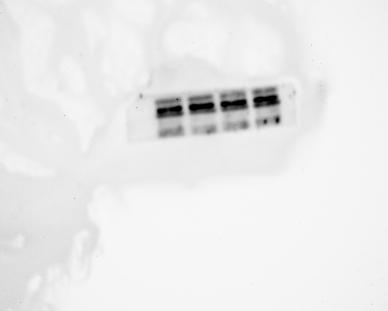

Supplement: Supplementary file 1 [file Supplementaryfile1.docx]
